# Visceral Fat Affects Heart Rate Recovery but Not the Heart Rate Response Post-Single Bout of Vigorous Exercise: A Cross-Sectional Study in Non-Obese and Healthy Participants

**DOI:** 10.3390/sports12120323

**Published:** 2024-11-27

**Authors:** Alessandra Amato, Luca Petrigna, Martina Sortino, Giuseppe Musumeci

**Affiliations:** 1 Department of Biomedical and Biotechnological Sciences, Section of Anatomy, Histology and Movement Science, School of Medicine, University of Catania, Via S. Sofia n°97, 95123 Catania, Italy; alessandra.amato@unict.it (A.A.); luca.petrigna@unict.it (L.P.); martinasortino97@gmail.com (M.S.); 2 Research Center on Motor Activities (CRAM), University of Catania, Via S. Sofia n°97, 95123 Catania, Italy

**Keywords:** adipose tissue, exercise physiology, body composition, heart rate recovery, exercise testing, training

## Abstract

Body composition could influence exercise physiology. However, no one has ever studied the effect of visceral fat (VF) on heart rate (HR) trends during and after exercise by using bioimpedance analysis (BIA). This study aims to investigate BIA variables as predictors of HR trends during vigorous exercise. Ninety-six participants (age 22.5 ± 4.8 years) were included in the data analysis. After performing BIA, the HR was recorded at three time points: baseline HR (BHR), peak HR (PHR) at the end of vigorous exercise, and resting HR (RHR) 1 min after the end of the exercise. After BHR, a 30 s squat jump test was performed. Linear regression analysis showed the body fat percentage and VF as a predictor of HR recovery post-exercise (*p* < 0.01). However, body weight has no association with HR recovery (*p* > 0.05). On the other hand, BIA variables were not associated with the variation of HR from the baseline to the end of the exercise. The results show that higher VF is associated with a slower HR recovery. To schedule a training program, it would be safer to monitor visceral fat before prescribing recovery time.

## 1. Introduction

The physiology behind heart rate (HR) variation during exercise is a key aspect to monitor during training in both professional and amateur athletes who approach exercise to maintain an active lifestyle. In recent years, the HR peak variation (HRPV) from baseline HR and HR recovery (HRR) post-exercise are becoming key fitness-related variables because they reflect changes in the combinations of parasympathetic withdrawal, sympathetic activation, and parasympathetic nervous system reactivation, respectively [1]. In addition, this cardiac health metric is usually also used for prognostic purposes; alternated HRR ability is associated with cardiovascular disease and mortality [2].

Several factors affect HRR and HRPV, including characteristics of the exercise stimulus, hydration, sleep quality, pathological conditions (hypertension, cardiac myopathies), physiological variables (age, gender, and circadian rhythm), environmental factors (stress, ambient temperature), lifestyle (physical activities, alcohol consumption, cigarette smoking, nutrition status), and non-modifiable factors such as genetic variables [3]. These factors affect the ability of the cardiovascular system to adequately adapt HR to different stimuli [4]. Indeed, sympathetic and parasympathetic performance vary in men or women up to the age of 50 and at different ages. Younger individuals have high HR variability up to the age of 15, while it decreases at older ages [5]; Bradley et al. demonstrate faster HRR in trained people compared with less trained people [3].

However, body composition variables as factors that possibly influence HRR and HRPV during exercise are poorly investigated with expensive, unaffordable tools and, just in elderly or pathological subjects, populations hard to evaluate in terms of confounding factors to find standardized reference values.

In addition, no studies consider visceral fat (VF) or total body fat (BF) as a predictor of HR variation during vigorous free-body exercise, including the amount of daily physical activity and exercise performance results in the same model. Understanding the role of these two variables in HR variation following a vigorous exercise could provide us with useful information to manage training schedules, especially in the choice of recovery time that is adequate for participants with special needs, such as high adipose tissue. VF is the fat located in the inner abdominal cavity stored deeper in the skin that surrounds the vital organs, including the heart [6]. An excessive amount can lead to certain diseases or dysfunctions affecting the cardiovascular system [7]. Previous studies have reported the possibility that VF influences exercise tolerance. It is known that VF accumulation can alter cardiovascular function by affecting the heart’s response to exercise. Chang et al. suggest that excess VF correlates with the autonomic response of HR to exercise, indicating increased pressure on the cardiovascular system [8]. The mechanisms of action known to date that explain the influence of VF on the autonomic nervous system are linked to the concentration of autonomic ganglia and axons, which modulate the HR in the VF in the epicardial adipose tissue; the modulation of these autonomic ganglia can induce cardiac arrhythmia, with additional potential for cardiovascular disease [8]. It has also been found that local inflammatory cytokines can affect the function of neighboring nerve ganglia of the autonomic system; inflammatory cytokines are known to modulate the excitability of ganglion neurons. Adiponectin and inflammatory cytokines secreted from visceral fats could explain the abnormality of cardiac autonomic response during exercise [9].

Indeed, Ramirez et al. found an association between HR variation during a maximal effort-limited exercise test with concentrations of inflammatory markers IL-6 but also with leptin, resistin, and insulin resistance (HOMA-IR), all markers related to high adipose tissue concentration [10]. Participants with a high tolerance for exercise and physical activities appear to have a lower visceral fat area [11].

However, VF is often investigated by expensive, invasive, or time-consuming methods, such as magnetic resonance imaging (MRI) [11,12] or (CT) scan [13], that can be found only in appropriate medical centers and whose use is not easy, especially in sports, and limited to the presence of a physician. VF is often analyzed using indirect methods such as waist/hip ratio [14], and these turn out to be inaccurate tests [15]. One method that might be a fair trade-off and that has been gaining ground in recent decades in the field of body composition assessments is bioimpedance analysis (BIA). Bioimpedance, through the deep penetration of very small electrical signals into the tissue, gives information that is modulated with blood flow, respiration, hydration, muscle contraction, and other physiological factors [16]. Body tissues are stimulated with low-amplitude and high-frequency electrical current and with a low-noise-sensing circuit. In the human body, fat, muscle, and vessels have different electrical properties, and the bioimpedance measurements are sensitive to the differences in the tissue composition [17,18,19]. This tool is easier to find than MRI in sports settings or in facilities that work to restore a healthy lifestyle in overweight people, such as gyms or dietitians’ offices, and can give clear and immediate information about body composition.

However, to date, the association between HRR and HRPV detected during and after a 1 min interval of vigorous exercise and body composition variables, including VF, collected by BIA has not been investigated in a young, healthy population. This study aims to investigate body composition variables as predictors of HR trends during and after 30 s of vigorous exercise, with a specific focus on VF’s effect on HRR. The second purpose is to detect this association for the first time by analyzing body composition parameters with BIA. This tool is cheaper and more easily accessible to professionals like trainers. Given the mechanisms of action known to date and described above in our research hypothesis, we expect participants with higher visceral fat to have slower HRR after exercise.

## 2. Methods

### 2.1. Study Design

**Figure 1:**
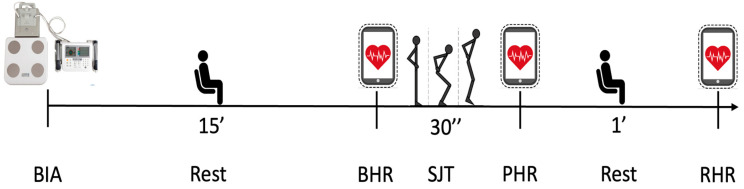
Study design and timeline. BIA: bioimpedance analysis; BHR: baseline heart rate; SJT: squat jump test; PHR: peak heart rate; RHR: resting heart rate.

Our study was a cross-sectional study. Each participant performed all the assessments in a single day: first, BIA was performed in the morning, and after that, baseline HR (BHR) was collected. After the first HR detection was finished, a squat jump test (SJT) was performed, and two other HR recordings were taken immediately after the exercise and one minute after the end of the exercise. All evaluations were carried out at the Research Center in Motor Activities (CRAM) at the University of Catania (Via Santa Sofia 97, 95123 Catania, Italy). The laboratory consisted of three rooms: one where the participants waited their turn, another where only the BIA was performed, and the last room where the HR recording and SJT were carried out. In this last room, the temperature was constantly controlled through the use of air conditioners. The recruitment and data collection phase began in November 2023 and ended in February 2024. A diagram of the study design can be found in Figure 1.

The study was designed and approved by the Research Center on Motor Activities (CRAM) Scientific Committee (Protocol n.: CRAM-49-2024, 29 January 2024). Participants provided voluntary informed consent, aligning with international bioethical standards as per the Declaration of Helsinki. Before the study, the protocols were presented, and an informational questionnaire was administered to participants to collect personal information, which was then processed anonymously. Participants provided their written consent before the start of the study, and they could withdraw from the study at any time.

### 2.2. Participants

A total of 112 participants were recruited from the lists of sports science students at the University of Catania (Italy). They were invited by email to attend the data collection days. The method used to select the sample was the probability sampling method, in which samples are selected based on availability and willingness to take part and through “Quota sampling”, which probably allows the recruitment of a tailored sample that is in proportion to some characteristic or trait of a population (e.g., young, healthy, athletic, and able to perform vigorous exercise, etc.). Before starting the assessment, we administered a self-reported questionnaire to collect health, personal, and everyday life information that allowed us to understand whether the participants met the criteria for inclusion.

Particularly with the questionnaire, we collected useful information to determine the weekly Physical Activity (wPA) minutes of each participant. wPA was collected and considered one of the variables that could influence performance.

### 2.3. Inclusion and Exclusion Criteria

Participants were included if they were more than 18 years old, injury-free, not obese (BMI < 30), and able to perform a physical test. They were excluded if they presented cardiac impairment, obesity, diabetes, hypercholesterolemia, hypercholesterolemia, eating disorders, lower limb injuries in the previous 6 months, disease of the locomotor system, or used not removable electronic devices or metallic objects. In addition, participants were excluded from data analysis if they did not achieve a vigorous effort, according to The American College of Sports Medicine guidelines [20], during the squat jump test.

### 2.4. Body Composition Assessment

**Figure 2:**
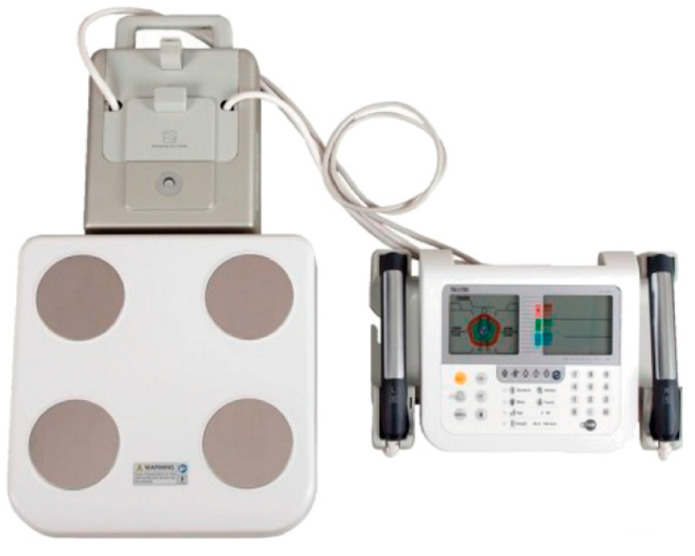
Picture of the bioimpedance meter “MC-780A TANITA” used for the study.

The tool used for the BIA was the MC-780A TANITA (TANITA Corporation, Tokyo, Japan) (Figure 2). This tool is demonstrated to accurately estimate body composition [13]. Participants reached the Research Center on Motor Activities of the University of Catania early in the morning after fasting from food and liquids for at least 8 h; overnight fasting was included, and breakfast was skipped by each participant.

Before the exam, we asked participants to remove any metal-type objects and step on the scale. Then, participants grasped the handles of the tool with their upper limbs extended without touching their thighs. Participants held this position for a few seconds to allow the tool to perform the analysis. During the examination, the participants were barefoot; male participants wore only shorts, and female participants wore shorts and a bra. Body composition variables were extrapolated from TANITA PRO software 2.0 by downloading for each participant as a Microsoft Excel © file. They included body weight (BW) in Kg, body fat percentage (BF), and visceral fat rating (VFr). VFr provided by TANITA scales is an estimate based on multiple factors, including electrical impedance, height, weight, age, and gender. The unit of measurement used by TANITA PRO software 2.0 for visceral fat is represented as an area (cm^2^). Each point on the TANITA visceral fat rating scale corresponds to 10 cm^2^ of visceral fat as estimated in comparison to MRI scans. The tool ranges the VF between 1 and 59. VFr was considered one of the body composition factors.

### 2.5. Heart Rate Recording

**Figure 3:**
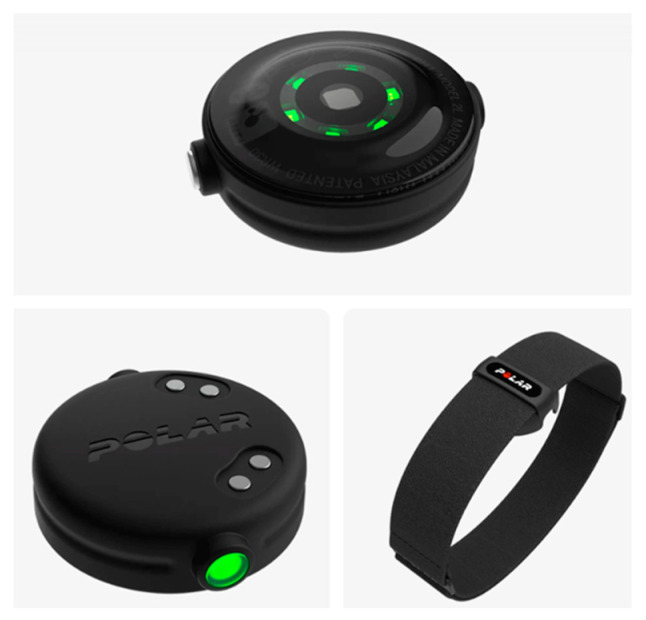
Picture of the heart rate monitor “Polar ^®^ OH1” used for the study.

The Polar ^®^ OH1 optical HR sensor from Polar ^®^ (Polar Electro Inc., Bethpage, NY, USA) was used for HR recording (Figure 3). The Polar ^®^ OH1 is a validated HR sensor [21]. The tool was worn on an armband on the lower or upper arm throughout the three readings and between the readings.

The sensor transmitted live data via Bluetooth ^®^ to compatible mobile devices (a tablet) where Polar’s applications (Polar ^®^ Flow) were installed. Participants were not allowed to see HR trends on the tablet. The OH1 records at 1 s intervals using 6 LED sensors. The HR was collected at three time points, as follows:

1. The first was the “baseline heart rate” (BHR), collected after 15 min of acclimatization in the same room where exercise was performed, with the participants sitting on a chair. The measurement was taken three times, and the mean value was taken into consideration for the data analysis.
2. The second detection was the “peak heart rate” (PHR), taken immediately after the end of the 30 s exercise.
3. The third was the “resting heart rate” (RHR); participants were helped to chairs, and the RHR detection was obtained after one minute of rest post-exercise.

### 2.6. Second Squat Jump Test

The 30 s SJT is a vigorous exercise ideal for expressing maximal muscle power [22], and it is a reliable evaluation [23].

Participants were asked to stand with their arms along their sides and their legs as wide as their shoulders. Then, after a start signal, the participants had to perform as many squat jumps as possible. The participants did not reach the full squat position necessary when they landed again, but the half squat position was enough to ensure the speed of execution of the exercise. This information was given to all participants before starting the test. The operator incited the participants to perform as many jumps as possible and reach as high as possible. Number of jumps (NJ) was collected and considered a “performance variable”. A timer with an audible signal was used to count the 30 s. According to The American College of Sports Medicine, vigorous exercise is defined as 60–89% of HR reserve [20]; for this reason, participants who did not reach 60% of HR as the effective intensity after 30 s of exercise were excluded from data analysis.

### 2.7. Data Analysis

For our data analysis, measurements were presented as mean (standard deviation). Descriptive statistics were computed for all the variables.

HRR was calculated as the difference between RHR and PHR (HRR = PHR − RHR), and HRPV was defined as the difference between PHR and BHR (HRPV = PHR − BHR) for each participant. Any negative values for HRR and HRPV present in the spreadsheet and identified before the data analysis were criteria for exclusion from the data analysis. Statistical assumptions for linear regression were verified as follows.

The normality of the distribution of the variables was verified using the Shapiro–Wilk test; Square Root Transformation was used to normalize dependent variables with not normal distribution; Homoscedasticity was verified through scatterplot of residuals analysis; multicollinearity between independent variables was tested through Variance Inflation Factor (VIF) calculation. In addition, relationships between the variables studied were determined using correlation analysis and the Pearson correlation coefficient. If statistically significant correlations were found, further relationships were studied to determine the size of the effect of the independent variable on the dependent variables. The relationships between the dependent variables (HRR and HRPV), the factors (VFr (cm^2^), BF (%), BW (kg)), and the possible confounding factors (wPA, NJ) were estimated using linear regression analysis. The a priori sample size of 92 was calculated with “G*Power” software (version 3.1.9.6) [24] performed with an effect size of 0.15, calculated from the previously published study with a similar study population [12].

Statistical analysis was performed with IBM SPSS statistics software (version 29.0.0.0, 241). The statistical significance level was set at *p* < 0.05 (two-tailed).

## 3. Results

**Figure 4:**
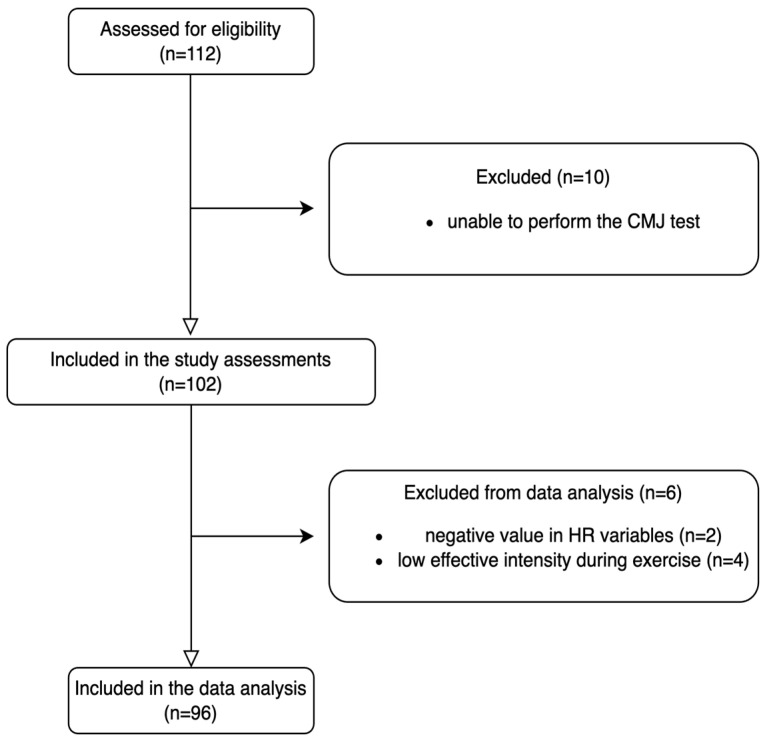
Flow chart representing the recruitment process.

Two participants’ results had negative values for HRR and HRPV on the spreadsheet, which led to their exclusion from the data analysis. In addition, four participants were excluded from the data analysis because they did not achieve a vigorous effective HR value according to our inclusion criterion (HR at the end of exercise > 60%) during exercise. Ninety-six participants (age 22.5 ± 4.8 years; height 169.7 ± 10.5 cm; body mass index 23.2 ± 3.7) were included in the data analysis: 40 females and 56 males. All participants were white ethnic Europeans (Figure 4).

The self-reported questionnaire shows that the entire sample had an average wPA of 252.3 ± 195.8 min. The average effective intensity reached by the participants immediately after the exercise was 74.8 ± 4.8% of the maximum HR. Participants performed an average NJ of 32.13 ± 5.6 during the SJT. Answers to the self-reported questionnaire are summarized in Table 1.

The descriptive analysis for each considered variable is shown in Table 2.

The normality check showed that one dependent variable (HRR) did not have a normal distribution of the data (Shapiro–Wilk, *p* < 0.05). Thus, Square Root Transformation (sqrt) was used to normalize HRR. The association between HR variables (HRR and HRPV), the factors (VFr (cm^2^), BF (%), BW (kg)), and the possible confounding factors (wPA, NJ) are shown using a correlation table (Table 3). In this study, Pearson’s correlation coefficient calculation method was used.

No evidence of association was found between HRPV and all three body composition factors or confounding factors (*p* > 0.05); on the other hand, an association was found between HRR, VFr, and BF. Therefore, multiple regression models were generated to examine the relationship between HRR and body composition variables.

Multiple regression analysis was performed using the wPA (min), NJ, and each body composition variable to confirm that multicollinearity did not occur.

The model was then created and analyzed using one selected body composition factor in addition to the possible confounding factors:

- Model 1 was generated using VFr (cm^2^) as the body composition factor.
- Model 2 was generated using BW (Kg) as the body composition factor.
- Model 3 was generated using BF (%)as the body composition factor.

**Figure 5:**
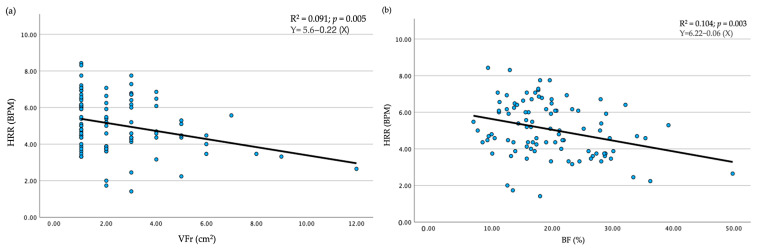
Regression variable plot. Dependent variable HRR was plotted against each variable, VFr (**a**) and BF (**b**), that resulted as significant predictors; In this scatter plot, each dot represents a participant tested HRR: heart rate recovery; VFr: visceral fat rating scale; BF: body fat percentage.

In all models, the coefficient of determination, R^2^, showed low values (R^2^ ≤ 0.1). However, while no significance was found for BW as an explanatory factor (*p* > 0.05; R^2^ < 0.1), VFr and BF are important explanatory factors among the body composition variables (VFr *p* = 0.005, R^2^ = 0.091; BF *p* = 0.003, R^2^ = 0.104) for HRR (Figure 5a,b), but the coefficient of determination, R^2^, also showed a low value. Both confounding factors, NJ and wPA, did not result as predictors of HRR (*p* > 0.05) (Table 4: a).

When we set HRPV as the dependent variable, no model seems to be predictive (*p* > 0.05; R^2^ < 0.1) (Table 4: b). The three models are summarized in Table 4.

## 4. Discussion

Our study aimed to investigate body composition variables as predictors of HR trends during and after vigorous exercise. Our results showed that the body composition variables VFr (*p* < 0.01) and BF (*p* < 0.01) but not BW (*p* > 0.05) are predictors for HRR but not for HRPV. Therefore, according to our hypothesis, participants with a higher VFr had a slower decrease in HRR in one-minute post-exercise. A previous study [12] showed an association between VF and exercise tolerance, demonstrating an association between VF and maximum oxygen uptake after a cardiopulmonary exercise test with a bicycle ergometer. In addition, it was shown that those with low cardiorespiratory fitness had a higher VF [25]. However, none of these studies analyzed the association between the VF rating scale and the exercise intensity detected by the HRR and HRPV trend after vigorous free-body exercise in a young, healthy population.

Increased sympathetic activity seems to be associated with higher visceral fat [26]. Similarly, impaired parasympathetic activity was associated with high-fat mass [27]. In contrast with our results, Nagashima et al. [28] showed that cardiopulmonary function markers are strong predictors of an improvement in HRR but not the metabolic parameters BW, BMI, BF, and VFr. However, Nagashima’s work was a longitudinal study where there was a training program administration; they assessed the correlation between change in body composition variables and change in HRR because of adaptation to exercise after the training period. Instead, our study was a single bout, and we included the volume of physical activity performed during the week and performance volume in the analysis as possible confounding factors that could influence the two variables of HRR and HRPV. However, in all three multiple regression models we conducted, neither wPA nor NJ were found to be predictors of HRR. Therefore, building this model for the first time could allow us to identify the association between HRR and VF and evaluate it during a single motor test that could be administered before starting a period of training aimed at weight loss. This could be useful in the future in reducing risk factors for adverse events during training sessions in a target population such as overweight individuals.

Brinkworth et al. found a good correlation between the change in HRR and the metabolic parameters BW, BMI, and waist circumference after a program that only involved dieting without any change in physical activity [29]. The authors did not investigate VFr. Therefore, since in our model, the BF was found to significantly (*p* < 0.01) predict HRR but not BW, it can be supposed that an important influence in this relationship is had by BF, particularly VFr, BW being a function of BF.

The mechanism explaining the relationship between BF and cardiovascular physiology during exercise is still not fully elucidated. Visceral adiposity and abdominal fat [25] are strongly associated with insulin resistance through the increased secretion of free fatty acids and a decreased secretion of adiponectin from visceral adipose tissue [30]. Elevated free fatty acid levels alter the autonomic system by the overactivation of the cardiac sympathetic nervous system [8]. Circulating adiponectin levels are lower in individuals with increased VF [31]. Adiponectin has insulin-sensitizing, anti-inflammatory, and anti-apoptotic properties and, thus, a protective role in cardiometabolic diseases [32].

It is demonstrated that they play a role in muscle regeneration activation with the suppression of proteolysis [33] and the regulation of the cardiac autonomic nervous system through favorable effects on endothelial function by the modulation of signaling cascades in cells of the vasculature and, thus, in vascular remodeling, which is one of the first adaptations to exercise [9]. These mechanisms could directly influence exercise adaptation through cardiovascular and muscle physiology.

There is evidence that weight loss brings increased activation of vagal tone [28]. Thus, those who start with a lower fat mass are hypothesized to have an efficient activation of the vagal tone, and this could explain a faster restoration of BHR in those who had a lower VFr in our study. This could explain why the correlation between the VFr and HR trend during physical activity was found only in the HRR phase and not with HRPV. In fact, during the HRR phase, vagal tone activation should prevail over sympathetic tone activation that occurs during the physical exertion response phase (HRPV); thus, the predictivity of VFr concerning good vagal tone functioning would be greater [1]. Vagal activity is responsible for the control of innumerable physiological regulatory processes, such as reduction in HR and the vasodilation/vasoconstriction of blood vessels. Reduced activation of vagal tone is also responsible for HR increase during exercise, and HRR after exercise is a consequence of vagal tone reactivation. In contrast to our results, Sheng-Hsiung Chang et al., in 2022, found a significantly inverted association between VF and HRPV after the Bruce exercise protocol was performed on a treadmill (β-coef.: −0.2, *p* = 0.014) at 80% of max HR. However, the discrepancy with the data reported by Cheng et al. may be explained by a higher exercise intensity than that used in our study, and only the epicardial adipose tissue periaortic fat was analyzed as a component of VF in an older population [8].

It has been demonstrated that the value of 1 min HRR immediately after exercise strongly predicts mortality [2]; for this reason, it is an important parameter to monitor during exercise. However, the body composition variables’ influence on this parameter has not been investigated. In our study, we chose a 1 min interval because the HRR post-1 min rest corresponds to the fast phase of HRR and characterizes a period in which there is an abrupt and rapid decrease in HR that then slows down in the next few seconds [34]; it is a simple-to-measure parameter that could be important to consider in exercise test interpretation, especially in participants with cardiovascular risk factors, like obesity, before scheduling a training plan. In addition, a one-minute or less interval is often chosen as an ideal recovery interval between exercise sets of training focused on fat loss that are administered to overweight people because of the intensity goal of the training [35]. However, from the association between the VFr scale and slower HRR, it is assumed that people with high VF need a longer recovery time. Consequently, our results suggest the importance of monitoring VF as well as total fat before drafting a training program aimed at weight loss, which often includes high intensity with short recoveries. Recovery times should be rethought, and more than 60 s should be considered in training protocols administered to participants with possible risk factors. In addition, our study showed that this association could be investigated with adequate accuracy with a less expensive tool, the BIA, which may be more readily available to trainers or experts in the field.

### 4.1. Limitations

This study has limitations. We could not prove any causal relationships between visceral fat accumulation and HR trend, and to date, no one has validated BIA exclusively to estimate VF. The sample was recruited from a cohort of students and may not be representative of the entire population. This limited the generalizability of the results due to a specific and homogeneous population; although one researcher explained in detail how to fill out the physical activity questionnaire, this could be a cause of potential bias because of the self-reported nature of the questionnaire. In addition, the large variability in minutes of physical activity performed per week and body composition among participants could be considered potential confounding variables because they could influence the HR response to vigorous exercise. In addition, HR recovery was assessed only post-1 min, so we cannot conclude that the recovery of subjects with higher VFr is slower even at longer recovery times.

### 4.2. Practical Applications and Challenges for the Future

Before scheduling a training plan, it would be good to perform a bioimpedance to assess any excess visceral fat and accordingly adapt the exercise to the needs of the participants, if necessary, by reducing the intensity by modulating the recovery time, which, from our results, should be more than 60 s if the exercise is vigorous (more than 60% of maximum heart rate). In addition, a simple 30 s vigorous free-body test appears to be adequate for investigating HRPV and HRR. However, future studies may aim to identify a specific threshold level of VF beyond which exercise intensity should be modulated because there may be insufficient HRR. In addition, it would be interesting to include other HR measurements at different periods and exercise intensities and involve populations with different body compositions, such as obese participants, or with different ages and underlying possible gender differences. In the end, it would be interesting to compare the results obtained from the BIA with those obtained from the gold-standard tests.

In conclusion, to our knowledge, the present study is the first to report an association between visceral fat rate evaluated by BIA and HR recovery 1 min post-exercise in a young, healthy population. HR recovery post-exercise is influenced by visceral fat but not by BW. HRPV is not influenced by body composition variables in a young, healthy population.

## Figures and Tables

**Table 1:** Self-reported questionnaire answers referring to participants’ PA habits.

Questions	Answers							
“Physical activity/sports practiced”	NO P.A	Strength training	Soccer	Volleyball	Basketball	Martial arts	Swimming	Other P.A
	10.1%	51.1%	10.1%	4.3%	4.3%	2.9%	0.7%	16.5%
“How many times per week?”	0 (day)	1 (day)	2 (day)	3 (day)	4 (day)	5 (day)	6 (day)	7 (day)
	1.4%	5.8%	15.8%	36%	20.9%	12.2%	6.5%	1.4%
“How long does an exercise session last?”	Less than 50 (min)	50 (min)	60 (min)	90 (min)	120 (min)	More than 120 (min)		
	2.9%	5%	16.5%	45.3%	23.7%	6.5%		
“How long have you been practicing it?”	Less than 6 months	6 months	1 (y)	2 (y)	3 (y)	4 (y)	5 (y)	More than 5 y
	20.3%	4.5%	9.8%	12.8%	12%	3.8%	2.3%	34.6%

P.A: physical activity; min: minutes; y: years.

**Table 2:** Descriptive analysis for body composition variables and HR variables for the entire sample. Data are expressed as mean and standard deviation (SD).

Variable	Value (Mean ± SD)
HRR (BPM)	27.8 ± 15.3
HRPV (BPM)	65.2 ± 19.6
BW (kg)	66.5 ± 11.1
BF (%)	19.7 ± 7.7
VFr (cm^2^)	2.4 ± 2.0

HRR: heart rate recovery; HRPV: heart rate peak variation; BPM: beats per minute; VFr: visceral fat rating scale; BF: body fat; BW: body weight.

**Table 3:** Correlation coefficients between HR variables (HRR + HRPV), body composition (VFr, BW, BF), and performance factor (wPA, NJ).

Variables	HRPV	HRR	BW (Kg)	VFr (cm^2^)	BF (%)	wPA (min)
HRR	0.250 *	--				
BW (Kg)	−0.093	−0.085	--			
VFr (cm^2^)	−0.047	−0.291 **	0.487 **	--		
BF (%)	−0.014	−0.305 **	0.053	0.543 **		
wPA (min)	0.85	0.001	0.188	0.061	−0.175	
NJ	−0.132	0.107	0.078	−0.094	−0.053	−0.027

*. Correlation is significant at the 0.05 level (2-tailed). **. Correlation is significant at the 0.01 level (2-tailed). HRR: heart rate recovery; HRPV: heart rate peak variation; VFr: visceral fat rating scale; NJ: number of jumps; wPA: weekly Physical Activity; BW: body weight; BF: body fat percentage.

**Table 4:** Summary of regression model predictors for HRR (a) and HRPV (b).

(a) Dependent Variable: HRR					(b) Dependent Variable: HRPV				
	Std. Error	β	*p*-Value	VIF		Std. Error	β	*p*-Value	VIF
Model 1					Model 1				
VFr (cm^2^)	0.076	−0.285	0.0005 **	1.012	VFr (cm^2^)	1.029	−0.065	0.531	1.012
NJ	0.027	0.080	0.423	1.009	NJ	0.363	−0.136	0.192	1.009
wPA (min)	0.001	0.021	0.833	1.004	wPA (min)	0.010	0.086	0.408	1.004
R^2^ = 0.091					R^2^ = 0.028				
Model 2					Model 2				
BF (%)	0.019	−0.310	0.003 **	1.035	BF%	0.266	−0.007	0.949	1.035
NJ	0.026	0.089	0.371	1.004	NJ	0.363	−0.130	0.210	1.004
wPA (min)	0.001	−0.050	0.617	1.033	wPA (min)	0.010	0.081	0.443	1.033
R^2^ = 0.104					R^2^ = 0.024				
Model 3					Model 3				
BW(Kg)	0.014	−0.099	0.352	1.044	BW (Kg)	0.185	0.103	0.329	1.044
NJ	0.028	0.115	0.270	1.008	NJ	0.362	−0.121	0.242	1.008
wPA (min)	0.001	0.023	0.827	1.038	wPA (min)	0.010	0.101	0.334	1.038
R^2^ = 0.021					R^2^ = 0.034				

** Correlation is significant at the 0.01 level (2-tailed). HRR: heart rate recovery; HRPV: heart rate peak variation; VFr: visceral fat rating scale; NJ: number of jumps; wPA: weekly Physical Activity; BW: body weight; BF: body fat percentage; VIF: Variance Inflation Factor.

## Data Availability

The data presented in this study are available on request from the corresponding author due to privacy or ethical restrictions.
